# Integration of Teaching of Digital Health-Driven Medical Devices in Pharmacy Education

**DOI:** 10.3390/pharmacy13020035

**Published:** 2025-03-01

**Authors:** Yasi Mojab, Eunjoo H. Pacifici, Terrence F. Graham, Rory E. Kim, Steven W. Chen

**Affiliations:** USC Alfred E. Mann School of Pharmacy and Pharmaceutical Sciences, University of Southern California, Los Angeles, CA 90089, USA; mojab@usc.edu (Y.M.); epacific@usc.edu (E.H.P.); grahamte@usc.edu (T.F.G.); rocallag@usc.edu (R.E.K.)

**Keywords:** medical devices, digital health, pharmacy education, interactive teaching, hands-on training

## Abstract

As medical devices become integral to modern healthcare, it is essential to prepare future pharmacists to counsel patients on device use and emerging therapeutic technologies. This study evaluates the impact of hands-on medical device training on pharmacy students at the University of Southern California (USC) Mann School of Pharmacy and Pharmaceutical Sciences, focusing on the level of comfort in counseling patients and retention of device-related information. Utilizing an active learning framework, this study provides insights into how experiential learning methods using medical devices enhance pharmacy students’ readiness for clinical practice. The results demonstrated significant improvement in levels of student comfort with counseling and information retention. The implementation of a hands-on training module has the potential to be adapted and applied to other courses or programs. The findings highlight the importance of integrating practical training within the pharmacy curriculum to better prepare graduates for effective patient education and support.

## 1. Introduction

With the rise of digital health technologies, companion diagnostics, and automated drug-delivery systems, it has become increasingly important for pharmacy students to gain practical experience in device handling and patient counseling [1]. Digital health tools include telemedicine, mobile health applications, wearable devices, and electronic health records [1]. These innovations enhance patient care by improving access, monitoring, and management. For example, automated insulin delivery systems, such as closed-loop insulin pumps, are significant advancements in diabetes management [2]. These systems automate insulin delivery based on continuous glucose monitoring, improving glycemic control and reducing patient burden. Pharmacists play a crucial role in educating patients about these devices, ensuring proper usage and troubleshooting issues [3]. Thus, practical training in these technologies is vital for pharmacy students to provide optimal diabetes care [4].

Pharmacy education is adapting to the evolving therapeutic landscape by incorporating digital health and medical devices into curricula to prepare students for technology-driven healthcare environments [1]. For instance, the International Pharmaceutical Federation (FIP) emphasizes developing a digitally enabled pharmaceutical workforce through comprehensive education on digital health applications [5]. Integrating active learning within medical education has been identified as essential for developing students’ competency and readiness for patient care [6]. Active Adult Learning (AAL) approaches enhance students’ engagement in patient-oriented settings, as demonstrated by improvements in affective responses to real-world applications [6,7].

Research findings underscore the importance of experiential, outcome-based curricula that bridge academic learning with practical application [1,2]. Such curricula are pivotal in equipping healthcare professionals to meet evolving patient care needs and demands. In pharmacy education, this approach fosters healthcare awareness at the community level and promotes interprofessional collaboration, an essential component in addressing complex healthcare challenges [1,5]. Additionally, integrating digital health competencies into pharmacy programs has become a pressing need. As healthcare systems increasingly adopt digital tools worldwide, pharmacy education must prepare students to leverage these technologies for enhanced patient-centered care. By blending digital literacy, hands-on training, and collaborative strategies, academic institutions can educate a workforce capable of adapting to and advancing in a dynamic healthcare environment [8].

To equip pharmacy students with the necessary skills, universities are integrating practical experiences with these technologies into their curricula [9,10,11]. This includes hands-on training with digital health tools, understanding the application of companion diagnostics, and managing automated medication delivery systems such as insulin. Such training ensures that future pharmacists are prepared to navigate and contribute to the evolving healthcare landscape [12].

Interactive learning and training are integral components of the Doctor of Pharmacy (PharmD) curriculum at USC Mann, where the evolving role of medical devices within therapeutic practice is emphasized [10,11]. These devices not only offer a specialized method of pharmaceutical delivery but are also approved through specific regulatory pathways and often require prior authorization at pharmacy sites before being dispensed to patients [13,14,15]. Thus, pharmacy students should be well-versed in the clinical, pharmaceutical, regulatory, and economic aspects of these tools to provide effective patient consultations and facilitate access to these devices [11].

To address these issues, USC Mann has incorporated multiple opportunities for students to become familiar with medical devices throughout the four-year PharmD curriculum as preparation for professional advanced pharmacy practice experiences (APPEs). This device training has been adapted and incorporated as part of an Objective Structured Clinical Examination (OSCE) for second- and third-year pharmacy students and as a hands-on project in the USC International Student Summer Program (ISSP) [10]. In this study, we evaluate the impact of hands-on medical device training on pharmacy students’ self-assessment, focusing on their level of comfort in counseling patients on device use and the retention of key information regarding these devices. We also determine whether this approach prepares students to educate patients on emerging therapies, including biologics, thereby supporting medication adherence through proper administration and storage guidance.

## 2. Materials and Methods

### 2.1. Students

The PharmD curriculum at USC Mann, in Los Angeles, CA, United States, incorporates a mandatory OSCE for third-year students prior to their APPEs. This OSCE evaluates students’ competency in patient counseling through realistic patient-interaction scenarios. The study utilized a convenience sampling method, including all students enrolled in the respective courses and programs during the study period. Participation was voluntary, and no exclusion criteria were applied beyond enrollment in the course. This approach ensured that the sample was representative of the students who received training on digital health-driven medical devices.

To simulate real-life situations, evaluators act as patients receiving a new medication or medical device, engaging the students in counseling on its proper use. PharmD students are presented with various medical device demonstrations and assessed during the OSCE to determine their proficiency levels. The curriculum has evolved over time: PharmD students who took the OSCE in 2023 (PharmD 2023 cohort) were introduced to medical devices during their third year, while PharmD students who took the OSCE in 2024 (PharmD 2024 cohort) were introduced to medical devices earlier, starting in their second year, with continued emphasis during their third-year OSCE. This progressive approach aims to enhance students’ skills and confidence in counseling patients on medical devices.

The participants in ISSP 2023 and ISSP 2024, advanced undergraduate Pharmacy students in their respective countries, collaborated within teams to explore pharmaceutical, clinical, and regulatory aspects of their designated device. Instead of the OSCE, ISSP 2023 and ISSP 2024 students displayed and presented their devices during a medical device trade fair simulation and were evaluated based on their presentations. The ISSP is a non-degree program in which students earn a certificate of completion at the end of the program. Presenting medical devices was a mandatory part of the program.

### 2.2. Sample Size Estimation

The study utilized a convenience sampling method, including all students enrolled in the respective courses and programs during the study period. Participation was voluntary. This approach ensured that the sample was representative of students who received training on digital health-driven medical devices. A total of 556 students were invited to participate in the pre-and post-surveys, consisting of 169 third-year PharmD students from the 2023 cohort, 188 third-year PharmD students from the 2024 cohort, 101 students from the ISSP 2023 cohort, and 98 students from the ISSP 2024 cohort. The inclusion criteria encompassed third-year PharmD students at the USC Mann School of Pharmacy who were enrolled in the OSCE, which incorporated hands-on training with medical devices, as well as students participating in the ISSP, who engaged with medical devices as part of their program experience. The only exclusion criterion was the non-enrollment of students in the specified pharmacy courses or the ISSP program.

### 2.3. Medical Devices

The medical devices (Table 1) were obtained as demo devices (no active ingredients) from the device manufacturers. The procurement of medical devices for the program was accomplished through direct outreach to pharmaceutical companies and their sales representatives. Some devices were provided to USC Mann by a local independent pharmacy that had them readily available. To secure devices from pharmaceutical companies, details of the program and curriculum were shared with their communications and public relations offices.

In some cases, internal contracts were required, which involved USC’s Chief Operating Officer to finalize agreements. Additionally, certain companies required a Charitable Contribution Agreement between USC Mann and the organization prior to the donation of medical devices. This process, which often involved multiple parties within both institutions, took several weeks to complete. For accountability, some companies also requested supporting documentation, such as photographs and written reports, to confirm that the donated devices were exclusively used for educational purposes. Other companies donated devices without requiring extensive documentation or agreements, emphasizing their support for educational initiatives.

### 2.4. Study Objectives and Surveys

The integration of medical devices into the pharmacy curriculum serves as an innovative teaching module designed to provide hands-on training to PharmD students. The survey questionnaires utilized in the ISSP and the PharmD program are shown in Table 2. The post-survey included a few additional questions designed to evaluate the effectiveness of integrating medical devices into the curriculum. These extra questions aimed to gather insights on the impact of hands-on medical device training on students’ learning outcomes, confidence in patient counseling, and overall preparedness for practical applications.

The objectives and rationale of medical device integration into pharmacy education are shown in Table 3. To examine these objectives, pre- and post-surveys were administered to third-year PharmD students in Spring 2023 and Spring 2024 and to ISSP participants in Summer 2023 and Summer 2024. All survey questionnaires are included in the Supplementary Material Table S1.

### 2.5. Statistical Analysis

Data from surveys are presented as the number of responses and percentages. Students that responded to the pre- and post-surveys were included in further analyses with differences analyzed by two-sided paired *t*-test. In these analyses, *p* < 0.05 was considered significant. All calculations were performed using Microsoft Excel. Confidence in patient counseling, assessed on a 10-point scale, significantly improved across all four cohorts, as demonstrated by a two-sided paired *t*-test. In the PharmD 2023 cohort (*n* = 91), confidence scores increased from 5.3 ± 2.1 pre-training to 7.9 ± 1.5 post-training (*p* < 0.001). Similarly, the ISSP 2023 cohort (*n* = 68) showed an increase from 6.0 ± 2.3 to 8.4 ± 1.2 (*p* < 0.001). In the PharmD 2024 cohort (*n* = 38), scores rose from 5.7 ± 2.4 to 7.2 ± 2.4 (*p* < 0.001), while the ISSP 2024 cohort (*n* = 69) demonstrated an improvement from 5.3 ± 2.2 to 8.2 ± 1.4 (*p* < 0.001). These findings indicate a statistically significant increase in confidence levels following the training, emphasizing the positive impact of medical device education on pharmacy students’ preparedness for patient counseling.

## 3. Results

### 3.1. Comfort Using Medical Devices

A summary of the results of the pre- and post-session surveys is shown in Table 4. In the PharmD 2023 cohort (*n* = 169), 127 students responded to the pre-survey and 128 to the post-survey. Of the respondents, 92 (72%) felt uncomfortable counseling patients on medical device use before the session. This decreased to 20 (16%) post-activity. In the PharmD 2024 cohort (*n* = 188), 132 students responded to the pre-survey and 48 to the post-survey (with the low response rate due to a conflict with other activities). Of the respondents, 60 (45%) felt uncomfortable counseling patients on medical device use before the session, and this decreased to 11 (23%) post-activity. Out of 101 participants in ISSP 2023, 90 responded to the pre-survey and 93 to the post-survey. Of these respondents, 45 (50%) felt uncomfortable counseling patients on medical device use pre-activity, which decreased to 4 (4%) post-activity. Out of 98 participants in ISSP 2024, 97 responded to the pre-survey and 78 responded to the post-survey. Of these respondents, 72 (74%) felt uncomfortable counseling patients on medical device use pre-activity, which decreased to 10 (13%) post-activity.

### 3.2. Confidence in Patient Counseling on Medical Devices

Over 95% of the participants from all cohorts agreed that pharmacy students should be trained on the use of medical devices. For respondents who answered both the pre- and post-surveys, confidence in patient counseling (assessed on a scale from 1 to 10) increased significantly in all three cohorts, as shown by a two-sided paired *t*-test: PharmD 2023 (*n* = 91, pre vs. post: 5.3 ± 2.1 vs. 7.9 ± 1.5, *p* < 0.001); ISSP 2023 (*n* = 68, pre vs. post: 6.0 ± 2.3 vs. 8.4 ± 1.2, *p* < 0.001); PharmD 2024 (*n* = 38, pre vs. post 5.7 ± 2.4 vs. 7.2 ± 2.4, *p* < 0.001); and ISSP 2024 (*n* = 69, pre vs. post 5.3 ± 2.6 vs. 8.2 ± 1.4, *p* < 0.001).

### 3.3. Changes in Student Comfort Levels in Patient Counseling

**Figure 1:**
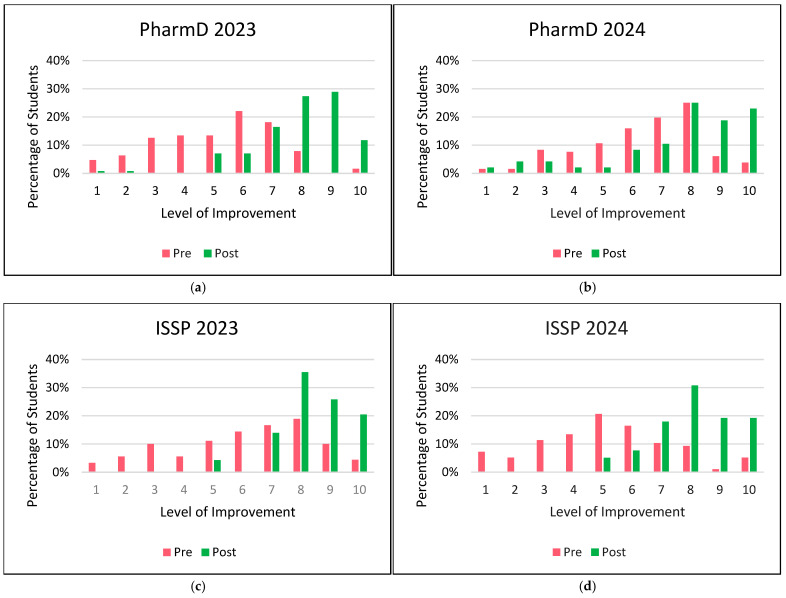
Comparison of student comfort levels for patient counseling pre vs. post activity. Pre-survey data are shown in pink, and post-survey data are shown in green. (**a**) PharmD 2023; (**b**) PharmD 2024; (**c**) ISSP 2023; (**d**) ISSP 2024.

Figure 1 compares pre- and post-activity comfort levels of students in patient counseling. All cohorts showed a substantial increase in comfort levels post-activity, particularly for higher comfort levels (7–10), with a shift from lower levels pre-activity (levels ≤ 6). In PharmD 2023 cohort, students’ comfort levels increased from 35 (28%) pre-activity to 108 (84%) post-activity. In PharmD 2024 cohort, students’ comfort levels increased from 72 (55%) pre-activity to 37 (77%) post-activity. In ISSP 2023 cohort, students’ comfort levels increased from 45 (50%) pre-activity to 89 (96%) post-activity. In ISSP 2024 cohort, students’ comfort levels increased from 25 (26%) pre-activity to 68 (87%) post-activity.

**Figure 2:**
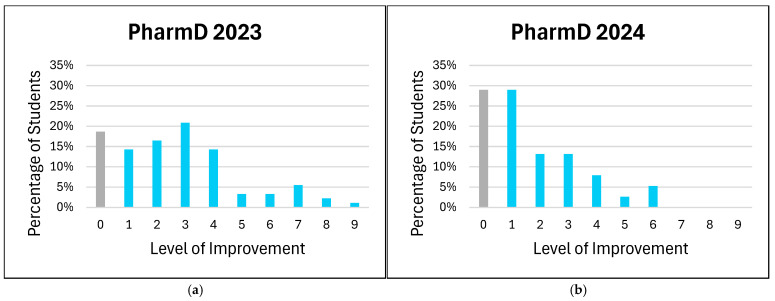

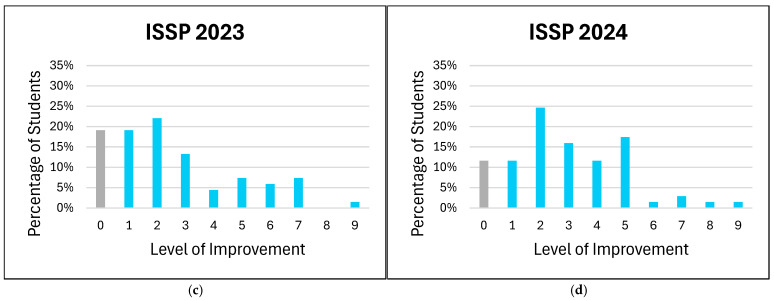
Level of improvement in students’ comfort level for patient counseling on medical devices. The degree of improvement is represented in blue, while no improvement is represented in gray. (**a**) PharmD 2023; (**b**) PharmD 2024; (**c**) ISSP 2023; (**d**) ISSP 2024.

The distribution of improvement levels in students’ comfort with patient counseling on medical devices is shown in Figure 2. In this analysis, all negative values representing non-improvement (i.e., negative changes in comfort level) were aggregated as a value of zero. This approach simplifies the interpretation by considering all instances where no improvement or a decline in comfort occurred as non-improvement. By grouping negative values with zero, we aimed to provide a clearer distinction between students who did and did not show improvement, without overemphasizing minor regressions in comfort levels.

### 3.4. Improvement in Information Retention

**Figure 3:**
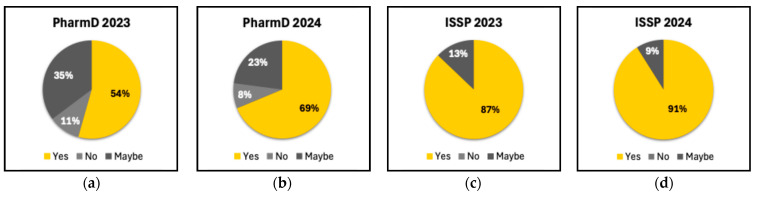
Students’ perception of the impact of medical devices on information retention compared to traditional teaching approaches. (**a**) PharmD 2023; (**b**) PharmD 2024; (**c**) ISSP 2023; (**d**) ISSP 2024.

Figure 3 illustrates the percentage of students from the four cohorts who believe that using medical devices enhances information retention when compared to traditional teaching methods. In the PharmD 2023 cohort, the majority of students (54%) answered “Yes”, indicating that they believe medical devices improve information retention; 35% were uncertain (“Maybe”); and a small proportion (11%) felt that medical devices did not offer an advantage over traditional teaching approaches. The PharmD 2024 cohort showed a stronger positive response, with 69% answering “Yes” to improved information retention through medical devices; a smaller percentage of students (23%) were unsure; and 8% believed there was no improvement. The ISSP 2023 cohort showed the highest confidence in the value of teaching with medical devices, with 87% responding “Yes” and only 13% responding “Maybe”. No students in this group responded “No”. Similar to ISSP 2023, in the ISSP 2024 cohort, a large majority (91%) responded “Yes”, supporting the use of medical devices for improved retention, with only 9% unsure. There were also no students who responded “No” in this cohort.

### 3.5. Learning Impact of Medical Devices

**Figure 4:**
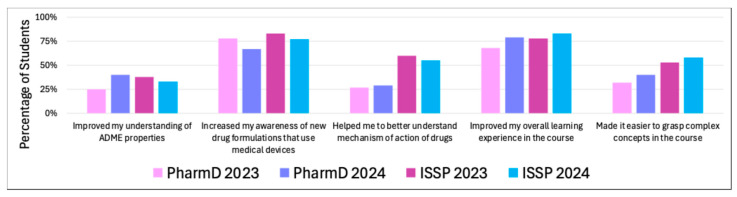
Percentages of students from the PharmD 2023, PharmD 2024, ISSP 2023, and ISSP 2024 cohorts who reported various learning impacts of use of medical devices in their education.

The impact of medical devices on learning (Figure 4) was assessed based on the following content in the post-survey (see Supplementary Tables S3 and S4):

Only 25% of PharmD 2023 students felt that medical devices helped improve their understanding of absorption, distribution, metabolism, and excretion (ADME) properties. Higher percentages were observed in the PharmD 2024 (40%), ISSP 2023 (38%), and ISSP 2024 (33%) cohorts, indicating a modest benefit across all groups.

Most students across all cohorts reported significant improvements in their awareness of new drug formulations that use medical devices: PharmD 2023 78%, PharmD 2024 67%, ISSP 2023 83%, and ISSP 2024 77%. This suggests that the use of medical devices was effective in raising awareness about drug formulations involving these technologies.

The improvement in understanding of the mechanism of action of drugs was moderate across cohorts: PharmD 2023 27%, PharmD 2024 29%, ISSP 2023 60%, and ISSP 2024 55%. The ISSP students seemed to benefit more in this area, whereas the PharmD students reported lesser impact.

A strong majority of students felt that medical devices improved their learning experience in the course: PharmD 2023 79%, PharmD 2024 68%, ISSP 2023 78%, and ISSP 2024 83%. The consistent high percentages suggest that integrating medical devices enhanced the overall educational experience for most students.

The impact of medical devices in simplifying complex concepts in the course was more varied. ISSP 2024 (58%) and PharmD 2024 (40%) students reported the most benefit, followed by ISSP 2023 (53%) and PharmD 2023 (32%). These results highlight that while a significant portion of students found devices helpful for understanding challenging topics, there was still a noticeable proportion who did not feel a strong impact in this area.

## 4. Discussion

This study underscores the importance of integrating hands-on medical device training into pharmacy education. Despite the growing role of medical devices in home healthcare and outpatient settings, many pharmacy students reported limited prior exposure to such devices, highlighting a critical gap in their training [1]. The integration of medical devices into both the ISSP and PharmD curricula provided students with practical skills and boosted their confidence in counseling patients on proper device use. This approach fostered better engagement and retention of course content compared to traditional didactic instruction and has potential for implementation in other curricula.

Student self-reported surveys indicated increased comfort in patient counseling following the training. While most students initially reported low-to-moderate comfort levels, post-activity surveys showed substantial increases, particularly at higher confidence levels of 7–10. Notably, the ISSP 2023 cohort exhibited the largest improvement at level 8, while the PharmD 2024 cohort showed pronounced increases at levels 8 and above. Variability in improvement among cohorts may be attributed to differences in initial comfort levels, exam-related anxiety (in the case of PharmD students), engagement with the training material, or pharmacy intern experience. Additionally, the PharmD 2024 cohort participated in another OSCE exam during their second year of pharmacy school that included medical devices component. This may have contributed to their results by providing additional practice in a clinical context.

The aggregated analysis of non-improvement (including zero and negative changes) revealed variability in the impact of the training across groups. For example, the PharmD 2023 cohort showed significant gains at moderate improvement levels, while the ISSP 2024 cohort demonstrated broader gains across both moderate and high levels. These findings suggest that targeted training can address diverse educational needs but may require further refinement to ensure consistent outcomes across cohorts. Students overwhelmingly recognized the value of medical device training in enhancing information retention, particularly among ISSP participants. However, the higher proportion of “Maybe” responses in the PharmD 2023 cohort may reflect varying familiarity or learning preferences. The absence of “No” responses among the ISSP groups may indicate robust support for integrating medical devices into pharmacy education or reflect limited hands-on training with clinical devices in certain international pharmacy schools.

Students reported an increased awareness of drug formulations and a better understanding of practical applications, with particularly positive responses from ISSP participants. While the benefits in understanding complex concepts were more moderate, especially among PharmD cohorts, these findings underscore the value of combining medical device training with complementary teaching methods for more comprehensive learning. Beyond clinical use, expanding the curriculum to include pharmaceutical sciences, regulatory considerations, and healthcare economics ensures a comprehensive understanding of the medical device life-cycle, from development to implementation. For example, courses at USC Mann in regulatory affairs and quality assurance enrich students’ perspectives on the scientific and regulatory processes that precede clinical trials. The interdisciplinary structure of the ISSP [10,11] further highlights the value of this approach. By assigning students to focus areas of clinical pharmacy, pharmaceutical sciences, or regulatory sciences, the program facilitates collaboration and provides exposure to diverse dimensions of medical device education [11]. This comprehensive training likely contributed to the high retention rates and positive feedback reported by ISSP participants.

Integrating medical device education aligns with the goals of pharmacy programs to enhance patient-centered care and prepare students for real-world clinical scenarios [13]. By placing this training in early foundational years, such as P1 or P2, students are better equipped for advanced practice settings. Feedback from participants emphasizes the importance of introducing hands-on training early in the curriculum to build preparedness and confidence. Moreover, this module serves as a scalable and adaptable approach that could be implemented in other educational programs, broadening its impact.

While this study focuses on the use of demonstration devices for hands-on learning in pharmacy education, similar principles can be applied to the integration of smart digital health technologies. Continuous glucose monitors, wearable devices, AI-based diagnostic tools, and mobile applications for disease management offer advanced data-driven insights that can enhance clinical decision-making and patient education [16]. Likewise, smart blood pressure monitors and other connected health devices provide real-time monitoring and personalized feedback, aligning with the growing role of pharmacists in digital health and remote patient management [16]. Incorporating these technologies into pharmacy curricula would not only expand students’ competency in emerging healthcare innovations but also prepare them for the evolving landscape of patient-centered, technology-driven care.

In the United States, digital health is incorporated into the 2025 Educational Outcomes and Activities established by the Accreditation Council for Pharmacy Education (ACPE) [17]. However, education on medical devices and their regulatory frameworks remains a distinct discipline and is not a mandated component of pharmacy curricula. Students seeking deeper knowledge in medical device regulations typically pursue specialized training beyond their PharmD, such as postgraduate fellowships in regulatory affairs or advanced degrees, including a Master of Science in Regulatory Science or relevant certifications [1].

Pharmacy education and practice vary globally, with differences in degree pathways and scope of practice. In the U.S., the Doctor of Pharmacy (PharmD) is the entry-level professional degree, granting graduates direct patient care responsibilities, including medication management, immunizations, and patient counseling. In contrast, other countries may offer Bachelor of Pharmacy (BPharm) or Master of Pharmacy (MPharm) degrees, with varied levels of clinical training and legal authority to prescribe or dispense medications [18]. These differences underscore the need for standardized yet adaptable medical device education, ensuring pharmacists worldwide are equipped with the necessary knowledge and skills to guide patients in device use, safety, and regulatory considerations.

This study has several limitations that must be acknowledged. First, the study was conducted at a single institution, which may limit the generalizability of the findings to other pharmacy programs. Second, the range of medical devices included in the educational activity was constrained by availability as only donated demo devices were incorporated into the curriculum. Future studies should consider expanding the variety of devices—including nebulizers, insulin pumps, and glucose monitors—to provide a more comprehensive learning experience. Additionally, while student confidence levels improved post-training, the observed increase was modest, potentially due to limited hands-on exposure, variability in prior knowledge, and time constraints of the training. A longitudinal study tracking students’ retention of knowledge and practical application of medical device counseling in clinical settings could provide deeper insights into the long-term impact of such education.

Despite these limitations, the findings highlight the importance of integrating medical device education into pharmacy curricula, particularly as pharmacists take on expanded roles in patient counseling and chronic disease management. Addressing these gaps through enhanced training programs and interdisciplinary collaboration could better prepare future pharmacists to navigate the evolving landscape of digital health and medical device integration.

This teaching methodology also has significant interdisciplinary potential. Expansion to medical, nursing, and allied health programs can promote collaborative learning and improve communication among healthcare professionals. The hands-on approach addresses the limitations of traditional teaching methods by actively engaging students and improving their ability to communicate complex device instructions to patients and coordinate care between disciplines. Future research should explore the long-term outcomes of such training on interdisciplinary collaboration and patient care. Expanding this model to other healthcare professions and international programs can bridge educational gaps and advance integrated, patient-focused healthcare systems.

## 5. Conclusions

Hands-on training with medical devices enhanced students’ retention of information and created a more active learning environment. Across all cohorts, students reported enhanced retention of information, greater preparedness for future careers, and improved confidence in patient counseling compared to traditional teaching methods. By fostering a more active learning environment, medical device training aligns seamlessly with the learning objectives of pharmacy curricula, emphasizing practical skills, patient-centered care, and interdisciplinary collaboration.

By incorporating medical device demonstrations into early foundational years (P1 or P2), students are likely to be better prepared for clinical rotations and professional practice. This hands-on approach equips them with the competence and confidence necessary to provide effective medication consultations and support patient adherence to prescribed regimens. Furthermore, the adaptability of the module makes it a valuable resource for other health professions, such as medical and nursing programs, fostering multidisciplinary collaboration and preparing future healthcare providers for real-world applications.

Overall, this study supports the integration of medical device training as a transformative addition to pharmacy education, ensuring students are well prepared to meet the evolving needs of patients and the healthcare industry.

## Figures and Tables

**Table 1:** List of medical devices used in PharmD and ISSP teaching.

Brand	Generic	Class	Medical Device Component ^a^
Trulicity	Dulaglutide	GLP-1 Receptor Agonist	Auto-injector pen
Evzio	Naloxone	Opioid Receptor Antagonist	Auto-injector trainer with voice instructions
Tymlos	Abaloparatide	Parathyroid Hormone Analog	Auto-injector with 4 needle pen components
Enbrel	Etanercept	Tumor Necrosis Factor Inhibitor	AutoTouch auto-injector
Baqsimi	Glucagon	Anti-hypoglycemic Agent	Nasal spray device
Lispro KwikiPen	Insulin	Insulin analogs	Prefilled insulin pen
Gvoke	Glucagon	Anti-hypoglycemic Agent	Injection HypoPen
Praluent	Alirocumab	PCSK9 Inhibitor	Auto-injector pen
Combivent	Ipratropium/Albuterol	Anticholinergic/Beta2-Agonist Combination	Metered-dose liquid inhaler
Benlysta	Belimumab	Monoclonal Antibody	Auto-injector
Tyrvaya	Varenicline	Alpha-4-Beta-2 nACh Agonist	Nasal spray
Eylea	Aflibercept	VEGF and PIGF Inhibitor	Pre-filled intravitreal injection
Lantus	Insulin Glargine	Long-acting Insulin	SoloStar push-button injector
Humira	Adalimumab	Tumor Necrosis Factor (TNF) Inhibitor	Auto-injector pen
Emgality	Galcanezumab	Antimigraine	Auto-injector pen

^a^ The demo version of these devices does not contain a functional needle for safety and demonstration purposes.

**Table 2:** Survey questions used in the ISSP and the PharmD program ^a^.

	Question	ISSP Pre	PharmD Pre	ISSP Post	PharmD Post
Q3	On the scale of 1–10, how comfortable are you counseling patients on the use of different medical devices (i.e., Insulin pens, auto injectors, etc.).	✓	✓	✓	✓
Q4	Have you used demo medical devices at work, or rotations?	✓	✓	✓	✓
Q5	Which area of study do you see integration of medical devices best fit? (Clinical Pharmacy, Pharmaceutical Sciences, Regulatory Science, Healthcare Economics)	✓	✓	✓	✓
Q6	In your opinion, should doctors of pharmacy, pharmacists, or healthcare workers get trained to use medical devices?	✓	✓	✓	✓
Q7	On the scale of 1–10, how beneficial would it be to include medical devices earlier in the PharmD. curriculum (i.e., P1 or P2 year)		✓		✓
Q8	Where do you see medical devices best fit in the PharmD curriculum?		✓		✓
Q9	Please name any other medical devices you would like to see.		✓		✓
Q10	Learning about medical devices… □ Improved my understanding of ADME properties □ Increased my awareness of new drug formulations that use medical devices □ Helped me to better understand mechanism of action of drugs □ Improved my overall learning experience in the course □ Made it easier to grasp complex concepts in the course			✓	✓
Q11	On a scale of 1–10, how helpful were the medical devices in enhancing your practical skills in the course?			✓	✓
Q12	Do you think the medical devices helped you to retain information better in comparison to traditional methods of teaching?			✓	✓
Q13	On a scale of 1–10, how comfortable were you in using the medical devices during the course?			✓	✓
Q14	Do you believe that the use of medical devices in the course helped you to be better prepared for your future career in the field?			✓	✓
Q15	Comment/Feedback			✓	✓

^a^ ✓ indicates that the question was asked in the particular program.

**Table 3:** Objectives and rationale for inclusion of medical devices in pharmacy teaching.

#	Study Objective	Rationale and Measurement	Survey Question
1	Enhance student confidence in counseling patients on medical devices.	Addressed by assessing comfort levels on a scale of 1–10 for counseling patients on medical devices (e.g., insulin pens and auto-injectors).	Q3
2	Increase exposure to hands-on use of demo medical devices.	Identified through survey responses regarding prior use of demo devices at work or during rotations.	Q4
3	Identify the most relevant areas of study for integrating medical devices.	Prioritized based on ranking areas of study where medical devices best fit, allowing focused curriculum integration.	Q5
4	Advocate for training healthcare students to use medical devices.	Supported by responses to the question on whether training in medical devices should be mandatory for pharmacy students.	Q6
5	Promote early introduction of medical device training in the PharmD curriculum.	Measured by responses on the perceived benefit of including medical devices earlier (e.g., P1 or P2 year).	Q7
6	Determine the optimal placement of medical device training within the curriculum.	Feedback on where students see medical devices best fitting in the PharmD curriculum helps align integration with curriculum design.	Q8
7	Improve practical skills through hands-on medical device training.	Assessed by a scale of how helpful medical devices were in enhancing practical skills during the course.	Post Q8
8	Enhance retention of information through the use of medical devices.	Evaluated by comparing retention of information using medical devices versus traditional teaching methods.	Post Q9
9	Prepare students for real-world applications in their future careers.	Feedback on whether the course helped students feel better prepared for their careers through medical device training.	Post Q8

**Table 4:** Results from surveys of students before and after the medical device session ^a.^

Q#	PharmD 2023 Pre	PharmD 2023 Post	PharmD 2024 Pre	PharmD 2024 Post	ISSP 2023 Pre	ISSP 2023 Post	ISSP 2024 Pre	ISSP 2024 Post
Q3 ^b^	92 (72%)	20 (16%)	60 (45%)	11 (23%)	45(50%)	4 (4%)	72 (74%)	10 (13%)
Q4 ^c^	40 (31%)	32 (25%)	26 (20%)	12 (25%)	21 (23%)	57 (61%)	26 (27%)	40 (51%)
Q5 ^d^	81 (64%),	87 (68%),	99 (75%),	36 (75%),	65 (72%),	80 (86%),	78 (80%),	68 (87%),
	21 (17%),	17 (13%),	14 (11%),	5 (10%),	9 (10%),	3 (3%),	6 (6%),	0 (0%),
	10 (8%),	11 (9%),	12 (9%),	6 (13%),	10 (11%),	8 (9%),	11 (11%),	7 (9%),
	15 (12%)	13 (10%)	7 (5%)	1 (2%)	6 (7%)	2 (2%)	2 (2%)	3 (4%)
Q6	121 (95%)	124 (97%)	128 (97%)	48 (100%)	89 (99%)	93 (100%)	95 (98%)	77 (99%)
Q7 ^b^	27 (21%)	19 (15%)	11 (8%)	2 (4%)				
Q8 ^e^	20 (16%),	13 (10%),	42 (32%),	16 (33%),				
	26 (20%),	28 (22%),	19 (14%),	7 (15%),				
	6 (5%),	3 (2%),	3 (2%),	0 (0%),				
	0 (0%),	1 (1%),	1 (1%),	1 (2%),				
	75 (59%)	83 (65%)	67 (51%)	24 (50%)				
Q10 ^f,g^		32 (25%),		19 (40%),		35 (38%),		26 (33%),
		100 (78%),		32 (67%),		77 (83%),		60 (77%),
		35 (27%),		14 (29%),		56 (60%),		43 (55%),
		87 (68%),		38 (79%),		73 (78%),		65 (83%),
		41 (32%)		19 (40%)		49 (53%)		45 (58%)
Q11 ^h^		98 (77%)		25 (52%)		90 (97%)		70 (55%)
Q12		68 (53%)		33 (69%)		81 (87%)		71 (91%)
Q13 ^h^		75 (59%)		34 (71%)		88 (95%)		70 (55%)
Q14		100 (78%)		38 (79%)		81 (90%)		71 (91%)

^a^ Response rates: PharmD 2023 Pre: 127/169 (75%), Post: 128/169 (76%); PharmD 2024 Pre: 132/188 (70%), Post: 48/188 (26%); ISSP 2023 Pre: 90/101 (89%), Post: 93/101 (92%); ISSP 2024 Pre: 97/98 (99%), Post: 78/98 (80%). ^b^ Percentage of students who ranked this question as ≤6 (Figure 1). Note that Q1 and Q2 are not shown because these questions collected demographic information. ^c^ Percentage of students who used demo devices. ^d^ Results shown in order of Clinical Pharmacy, Pharmaceutical Sciences, Regulatory Science, and Healthcare Economics. ^e^ Results shown in order of P1, P2, P3, P4, and Integrated through the PharmD curriculum. ^f^ Q9 and Q15 were free-response comment boxes, please see Supplementary Materials Table S2. ^g^ Percentage of students selecting each response option for the multiple-choice question on the impact of learning in the following order: improved my understanding of ADME properties, increased my awareness of new drug formulations that use medical devices, helped me to better understand mechanism of action of drugs, improved my overall learning experience in the course, and improved my overall learning experience in the course. ^h^ Percentage of students who ranked this question as ≥7.

## Data Availability

Data are available on reasonable request to the corresponding author. Please note that participating students name and email address will be kept confidential.

## Supplementary Materials

The following supporting information can be downloaded at https://www.mdpi.com/article/10.3390/pharmacy13020035/s1. Table S1: ISSP 2023 and ISSP 2024 pre-activity survey questions; Table S2: PharmD 2023 and 2024 pre-activity survey questions; Table S3: ISSP 2023 and ISSP 2024 post-activity survey questions; Table S4: PharmD 2023 and 2024 post-activity survey questions.
