# Supplementary material for: Integration of Teaching of Digital Health-Driven Medical Devices in Pharmacy Education
Source: Pharmacy (Basel). 2025 Mar 1;13(2):35. doi: 10.3390/pharmacy13020035 (PMC11932268; doi:10.3390/pharmacy13020035)
Supplement: Supplementary file 1 [file pharmacy-13-00035-s001.zip › pharmacy-3482393-supplementary.pdf]

Supplementary Material

Integration of Teaching of Digital Health-Driven Medical Devices in Pharmacy Education

Yasi Mojab, Steven W Chen, Eunjoo H Pacifici, Terrence F Graham, Rory E Kim, Ian S Haworth

Table S1. Questionnaire for USC International Student Summer Program (ISSP) PRE-Course Survey.

	Question	Question type
1	Name (Last, First)	Short answer/free response
2	Email	Short answer/free response
3	On the scale of 1-10, how comfortable are you counseling patients on the use of different medical devices (i.e. Insulin pens, auto injectors, etc.). (1 uncomfortable, 10 very comfortable).	Numerical Scaling answer
4	Have you used demo medical devices at work, or rotations?<br>☐ Yes<br>☐ No	Multiple choice
5	Which area of study do you see integration of medical devices best fit? Rank from 1-4 (1 being best fit and 4 being least fit).<br>Clinical Pharmacy<br>Pharmaceutical Sciences<br>Regulatory Science<br>Healthcare Economics	Numerical ranking choice
6	In your opinion, should doctors of pharmacy, pharmacists, or healthcare workers get trained to use medical devices?<br>☐ Yes<br>☐ No	Multiple choice

Table S2..Questionnaire for USC Doctor of Pharmacy (PharmD) PRE-Course Survey.

	Question	Question type
1	Name (Last, First)	Short answer/free response
2	Email	Short answer/free response
3	On the scale of 1-10, how comfortable are you counseling patients on the use of different medical devices (i.e. Insulin pens, auto injectors, etc.). (1 uncomfortable, 10 very comfortable).	Numerical Scaling answer
4	Have you used demo medical devices at work, or rotations?	Multiple choice (Yes, No)
5	Which area of study do you see integration of medical devices best fit? Rank from 1-4 (1 being best fit and 4 being least fit).<br>Clinical Pharmacy<br>Pharmaceutical Sciences<br>Regulatory Science<br>Healthcare Economics	Numerical ranking choice
6	In your opinion, should Doctor of Pharmacy get trained to use medical devices?<br>☐ Yes<br>☐ No	Multiple choice

7	On the scale of 1-10, how beneficial would it be to include medical devices earlier in the PharmD. curriculum (ie. P1 or P2 year). (1 NOT beneficial, 10 very beneficial)	Numerical Scaling answer
8	Where do you see medical devices best fit in the PharmD curriculum?<br>☐ P1<br>☐ P2<br>☐ P3<br>☐ P4<br>☐ Integrated throughout the PharmD curriculum	Multiple choice
9	Please name any other medical devices you would like to see.	Short answer/free response

Table S3. Questionnaire for USC International Student Summer Program POST-Course Survey.

	Question	Question type
1	Name (Last, First)	Short answer/free response
2	Email	Short answer/free response
3	On the scale of 1-10, how comfortable are you counseling patients on the use of different medical devices (i.e. Insulin pens, auto injectors, etc.). (1 uncomfortable, 10 very comfortable).	Numerical Scaling answer
4	Have you used demo medical devices at work, or rotations?	Multiple choice (Yes, No)
5	Which area of study do you see integration of medical devices best fit? Rank from 1-4 (1 being best fit and 4 being least fit).<br>Clinical Pharmacy<br>Pharmaceutical Sciences<br>Regulatory Science<br>Healthcare Economics	Numerical ranking choice
6	In your opinion, should doctors of pharmacy, pharmacists, or healthcare workers get trained to use medical devices?	Multiple choice (Yes, No)
7	Learning about medical devices…<br>☐ Improved my understanding of ADME properties<br>☐ Increased my awareness of new drug formulations that use medical devices<br>☐ Helped me to better understand mechanism of action of drugs<br>☐ Improved my overall learning experience in the course<br>☐ Made it easier to grasp complex concepts in the course	Select all that apply
8	On the scale of 1-10, how helpful were the medical devices in enhancing your practical skills in the course? (1 NOT helpful, 10 VERY helpful).	Numerical Scaling answer
9	Do you think the medical devices helped you to retain information better in comparison to traditional methods of teaching?<br>☐ Yes<br>☐ No<br>☐ Maybe	Multiple choice
10	On the scale of 1-10, how comfortable were you in using the medical devices during the course? (1 uncomfortable, 10 Very comfortable)	Numerical Scaling answer
11	Do you believe that the use of medical devices in the course helped you to be better prepared for your future career in the field?<br>☐ Yes<br>☐ No<br>☐ Maybe	Multiple choice

12	Please name any other medical devices you would like to see in the future ISSP program?	Short answer/free response
13	Comment/feedback	Short answer/free response

Table S4. Questionnaire for USC Doctor of Pharmacy (PharmD) POST-Course Survey.

	Question	Question type
1	Name (Last, First)	Short answer/free response
2	Email	Short answer/free response
3	On the scale of 1-10, how comfortable are you counseling patients on the use of different medical devices (i.e. Insulin pens, auto injectors, etc.). (1 uncomfortable, 10 very comfortable).	Numerical Scaling answer
4	Have you used demo medical devices at work, or rotations?	Multiple choice (Yes, No)
5	Which area of study do you see integration of medical devices best fit? Rank from 1-4 (1 being best fit and 4 being least fit).<br>Clinical Pharmacy<br>Pharmaceutical Sciences<br>Regulatory Science<br>Healthcare Economics	Numerical ranking choice
6	In your opinion, should Doctor of Pharmacy get trained to use medical devices?	Multiple choice (Yes, No)
7	On the scale of 1-10, how beneficial would it be to include medical devices earlier in the PharmD. curriculum (ie. P1 or P2 year). (1 NOT beneficial, 10 very beneficial)	Numerical Scaling answer
8	Where do you see medical devices best fit in the PharmD curriculum?<br>☐ P1<br>☐ P2<br>☐ P3<br>☐ P4<br>☐ Integrated throughout the PharmD curriculum	Multiple choice
9	Please name any other medical devices you would like to see.	Short answer/free response
10	Learning about medical devices…<br>☐ Improved my understanding of ADME properties<br>☐ Increased my awareness of new drug formulations that use medical devices<br>☐ Helped me to better understand mechanism of action of drugs<br>☐ Improved my overall learning experience in the course<br>☐ Made it easier to grasp complex concepts in the course	Select all that apply
11	On the scale of 1-10, how helpful were the medical devices in enhancing your practical skills in the course? (1 NOT helpful, 10 VERY helpful).	Numerical Scaling answer
12	Do you think the medical devices helped you to retain information better in comparison to traditional methods of teaching?<br>☐ Yes<br>☐ No<br>☐ Maybe	Multiple choice
13	On the scale of 1-10, how comfortable were you in using the medical devices during the course? (1uncomfortable, 10 Very comfortable)	Numerical Scaling answer
14	Do you believe that the use of medical devices in the course helped you to be better prepared for your future career in the field?	Multiple choice

	☐ Yes<br>☐ No<br>☐ Maybe	
5	Comments/Feedback	Free response
